# GSKIP modulates cell aggregation through EMT/MET signaling rather than differentiation in SH-SY5Y human neuroblastoma cells

**DOI:** 10.1007/s12079-023-00752-z

**Published:** 2023-05-03

**Authors:** Cheng-Yu Tsai, Huey-Jiun Ko, Shean-Jaw Chiou, Xin-Yi Lin, Tsung-Hsien Chuang, Jiin-Tsuey Cheng, Yu-Feng Su, Joon-Khim Loh, Yi-Ren Hong

**Affiliations:** 1 Division of Neurosurgery, Department of Surgery, Kaohsiung Medical University Hospital, Kaohsiung, Taiwan; 2 Post Baccalaureate Medicine, College of Medicine, Kaohsiung Medical University, Kaohsiung, Taiwan; 3 Graduate Institute of Medicine, College of Medicine, Kaohsiung Medical University, Kaohsiung, 807 Taiwan; 4 Department of Biochemistry, Faculty of Medicine, College of Medicine, Kaohsiung Medical University, Kaohsiung, 807 Taiwan; 5 Department of Medical Research, Kaohsiung Medical University Hospital, Kaohsiung, 807 Taiwan; 6 Immunology Research Center, National Health Research Institutes, Miaoli, 350 Taiwan; 7 Department of Biological Sciences, National Sun Yat-Sen University, Kaohsiung, 804 Taiwan; 8 Center for Cancer Research, Kaohsiung Medical University, Kaohsiung, 807 Taiwan; 9 Neuroscience Research Center, Kaohsiung Medical University, Kaohsiung, 807 Taiwan

**Keywords:** GSKIP, Cell aggregation, Proliferation, EMT/MET, Phosphor-β-catenin, Wnt/β-catenin

## Abstract

GSK3β interacting protein (GSKIP) is a small A-kinase anchor protein previously reported to mediate the N-cadherin/β-catenin pool for differentiation in SH-SY5Y cells through overexpression of GSKIP to present the neuron outgrowth phenotype. To further investigate how GSKIP functions in neurons, CRISPR/Cas9 technology was utilized to knock out GSKIP (GSKIP-KO) in SH-SY5Y. Several GSKIP-KO clones resulted in an aggregation phenotype and reduced cell growth without retinoic acid (RA) treatment. However, neuron outgrowth was still observed in GSKIP-KO clones treated with RA. The GSKIP-KO clones exhibited an aggregation phenotype through suppression of GSK3β/β-catenin pathways and cell cycle progression rather than cell differentiation. Gene set enrichment analysis indicated that GSKIP-KO was related to epithelial mesenchymal transition/mesenchymal epithelial transition (EMT/MET) and Wnt/β-catenin/cadherin signaling pathways, suppressing cell migration and tumorigenesis through the inhibition of Wnt/β-catenin mediated EMT/MET. Conversely, reintroduction of GSKIP into GSKIP-KO clones restored cell migration and tumorigenesis. Notably, phosphor-β-catenin (S675) and β-catenin (S552) but not phosphor-β-catenin (S33/S37/T41) translocated into the nucleus for further gene activation. Collectively, these results suggested that GSKIP may function as an oncogene to form an aggregation phenotype for cell survival in harsh environments through EMT/MET rather than differentiation in the GSKIP-KO of SH-SY5Y cells.

**Graphical abstract:**

**Figure:**
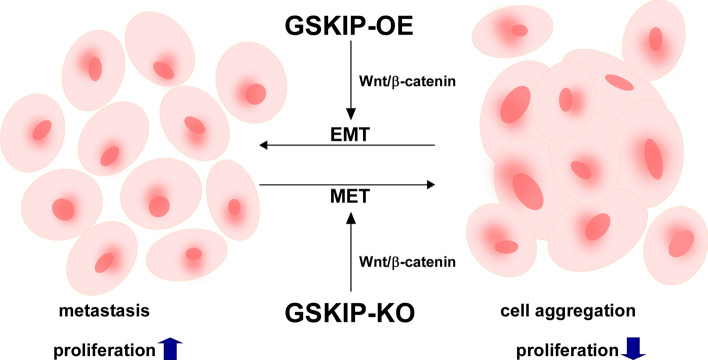
GSKIP Implication in Signaling Pathways with Potential Impact on SHSY-5Y Cell Aggregation

## Introduction

Glycogen synthase kinase interaction protein (GSKIP) is the smallest A-kinase anchor protein (AKAP) (Hundsrucker et al. 2010; Skroblin et al. 2010) originally cloned and identified as a glycogen synthase kinase-3β (GSK3β) interacting protein (Chou et al. 2006; Howng et al. 2010; Pilot-Storck et al. 2010; Tang et al. 2011). GSKIP is a cytosolic scaffolding protein that retains protein kinase A (PKA) RII-binding sites at the N-terminal residue V41/L45 and a GSK3β-binding domain at the C-terminal residue L130 (Chou et al. 2006; Cole et al. 2004; Howng et al. 2010; Lin et al. 2009; Tang et al. 2011). Furthermore, the GSK3β-binding domain is reported to act synergistically with cAMP/PKA signaling to inhibit GSK3β activity through a cytoplasmic destruction complex with β-catenin, leading to the negative regulation of Wnt signaling (Dema et al. 2016; Loh et al. 2015). GSKIP can stabilize β-catenin by directly competing with GSK3β binding for the phosphorylation at Ser675 by PKA and can destabilize β-catenin by directly competing with GSK3β binding for the phosphorylation at Ser-33/Ser-37/Thr-41 (Chou et al. 2018).

Increasing evidence has shown that GSKIP plays a variety of roles in the development of various diseases and cancers. GSKIP can directly modulate GSK3β activity to inhibit palatal shelf fusion in mice during embryonic development (Deák et al. 2016) and is associated with a predisposition to familial myeloid malignancies (Duployez et al. 2016; Plo et al. 2015; Saliba et al. 2015). GSKIP is also responsible for the PPARγ-related angiogenic potential of endothelial pulmonary microvascular endothelial cells (Vattulainen-Collanus et al. 2016). Recent studies have demonstrated that GSKIP could be regulated by miR-150-5p and was downregulated by different EGFR mutations in non-small-cell lung cancer cells (Dai et al. 2019; Nishimura et al. 2020). Moreover, GSKIP can also be upregulated by miRNA-218 and miR-181c-5p in small-cell lung cancer cells (Loh et al. 2015) and cervical squamous cell carcinoma (Li et al. 2020).

Recently, our group focused on the neuronal aspect of GSKIP, demonstrating that GSKIP has different critical roles in the neuronal system, as evident in neuron development, neuron protection, and neuro-degenerative diseases. We clarified that GSKIP has a role in the neuron development function through the transfection of the neuronal-like SH-SY5Y cell line with wildtype (WT) GSKIP to inhibit neurite outgrowth (Lin et al. 2009). We also demonstrated that GSKIP could modulate dynamin-related protein 1 (Drp1) phosphorylation to provide neuron protection against oxidative stress for SH-SY5Y cells, indicating that GSKIP functions as an anchor protein in the cAMP/PKA/Drp1 signaling axis (Loh et al. 2015). This protein PKA/GSKIP/GSK3β axis is involved in Tau phosphorylation for neurodegenerative diseases, such as Alzheimer’s disease (Ko et al. 2019). In addition, GSKIP was investigated using bioinformatics tools, site-directed mutagenesis, and yeast two-hybrid methods and eventually classified into simple-form and composite-form variants in an evolutionary context (Chou et al. 2018; Tsai et al. 2022; Wu et al. 2022).

This study continued to investigate how GSKIP functions in neuron outgrowth. Compared to our previous finding that GSKIP mediated the N-cadherin/β-catenin pool in the differentiation of SH-SY5Y cells through the overexpression (OE) of GSKIP, we utilized CRISPR/Cas technology to knock out GSKIP in SH-SY5Y to investigate whether GSKIP functions in cell differentiation or proliferation. Surprisingly, three GSKIP-KO cell clones resulted in an aggregated phenotype and reduced cell growth relative to GSKIP WT. Bioinformatics analysis tools indicated that GSKIP-KO was related to the epithelial mesenchymal transition/mesenchymal epithelial transition (EMT/MET) phenotype and highly associated with the Wnt/β-catenin/cadherin signaling pathway. Also, EMT was mediated by the critical mechanisms through which GSKIP-KO suppressed cell migration and tumorigenesis of SH-SY5Y cells via inhibition of Wnt/β-catenin. Furthermore, the phosphorylation of β-catenin, notably phosphor-β-catenin (S675) and β-catenin (S552), are involved in the translocation into the nucleus and subsequent downstream gene activation and upregulation of N-cadherin for N-cadherin-mediated β-catenin pools in the context of GSKIPs. Finally, GSKIP may function as an oncogene to aid cell survival in harsh environments through the formation of aggregation phenotypes, rather than differentiation through EMT/MET signaling in the GSKIP-KO of SH-SY5Y human neuroblastoma cells.

## Materials and methods

### Cell culture and differentiation of SH-SY5Y cells

The human neuroblastoma SH-SY5Y cell line was purchased from the American Type Culture Collection (ATCC, CRL-2266) and cultured in DMEM/F12 medium (Gibco; Thermo Fischer Scientific, Grand Island, NY, USA) supplemented with 10% fetal bovine serum (FBS, Gibco) and 1% streptomycin/penicillin (Gibco) at 37 °C with 5% CO_2_. When cells were 40–50% confluent, differentiation was initiated by the addition of 10 µM all-trans-retinoic acid (RA; Sigma) for 6 days. This treatment was replaced every 2 days to replenish RA in the culture media and the cells were analyzed by phase-contrast light microscopy. Images were captured with NIS-elements software.

### CRISPR/Cas9-mediated gene editing

To generate GSKIP deficient GSKIP-KO cells, CRISPR/Cas9-mediated gene (RGEN, RNA-Guided Endonuclease) editing was performed. Both strands of oligo DNAs encoding for one gRNA that specifically targets the GSKIP exon 2 sequences (sgRNA: 5′-CTCGAAAAGCCTGCGGTGTGCGG-3′, Fig. 2) were designed using an online CRISPR design tool (http://crispr.mit.edu/) provided by the Biotools company (Biotools, Madrid, Spain). The plasmids were transfected into the cells using TOOLSFect Transfection Reagent (Biotools) according to the manufacturer’s instructions. For the CRISPR/Cas-assisted gene, cells were transfected with the plasmids Cas9-plasmid (pRGEN-Cas9-CMV), surrogate-reporter, and gRNA-plasmid (pRGEN-Human-GSKIP), then 48 h after transfection, the cells were treated with 2 μg/ml of puromycin for three days. Dissociated cells were collected and resuspended in Dmem/F12 medium and then analyzed and sorted with a FACS Aria II (Becton Dickinson, Heidelberg, Germany). Enrichment of cells with RGEN activity was accomplished by single-cell sorting of HA epitope-tagged positive cells into 96-well plates. The colonies were isolated with the cloning cylinders, and the GSKIP sequences were analyzed with a T7 endonuclease (T7E1) assay report, F-PCR report, DNA sequencing, and western blotting (T7E1 assay report, F-PCR report, and DNA sequencing provided by Biotools). All experimental procedures were conducted following the manufacturer’s protocol, and mutated nucleotides were verified by DNA sequencing with an ABI PRISM 3730 Genetic Analyzer (Applied Biosystems, Forster City, CA, USA).

### Transfection

For transient transfections, approximately 5 × 10^4^ or 1 × 10^6^ cells were plated into 24-well plates or 100 mm dishes and left to grow overnight. The following day, vehicle (pEGFP) and pEGFP-GSKIP plasmid DNA (2 μg) were transfected into the cells using Lipofectamine 3000 (Invitrogen Corporation, CA, USA) according to the manufacturer’s instructions. After 48 h, the cells were cultured in fresh medium and further assayed.

### RNA isolation and quantification of target mRNA expression by TaqMan qPCR

Total RNA was extracted using RNA Isolater Total RNA Extraction Reagent (Vazyme, CN) and. quantified using an Epoch spectrophotometer with a Take3 plate (BioTek, US). Then, 1,000 ng of total RNA was reverse transcribed using a HiScript III RT SuperMix for qPCR (+ gDNA wiper) (#R323; Vazyme) according to the manufacturer’s instructions. The cDNA was amplified using the TopQ Gene Probe Assay for human GAPDH, GSKIP, and GSKIP-CRISPR (#600250, Topgen Biotech., TW; Primers and probes listed in Table S1, respectively). Quantitative PCR was performed with ChamGE Probe qPCR Master Mix (#CGE-01; Topgen Biotech., TW) in a 10 µL reaction on the StepOne Plus Real-Time PCR System (Applied Biosystems, USA) as per the manufacturer’s protocol. Then, 10 ng of cDNA was amplified in triplicate with the appropriate non-template controls. Amplification data were normalized to GAPDH expression and quantified using the 2-∆∆Ct method. Quantitative PCR data showed a variability coefficient of Ct always lower than 2% of mean values.

### Western blotting

Protein was extracted using RIPA lysis buffer (EMD Millipore Billerica, MA, USA, 10 × RIPA buffer) and quantified via the Bio-Rad DC protein assay (Bio-Rad, Hercules, CA). The protein samples were mixed with SDS loading buffer and separated on an 8–12% SDS-PAGE gel and transferred to PVDF membrane (NEF1002001PK, Perkin Elmer, Waltham, MA, USA). The membrane was then blocked with 5% BSA (Sigma) at room temperature for 1 h in Tris-buffered saline-Tween 20 (0.5%; TBS-T). After washing in TBS-T, membranes were incubated overnight at 4 °C in 2.5% BSA with the appropriate primary antibodies and then incubated with horseradish peroxidase (HRP)-conjugated secondary antibodies at room temperature for 1 h. All antibodies and dilutions are shown in Table S2. The immunoblot signals were developed using the Super Signal Ultra chemiluminescent reagent (Pierce, Rockford, US) and detected by a CCD camera (MultiGel-21, Topbio, Taipei, Taiwan).

### cDNA sequencing analysis by next-generation sequencing (NGS)

Total RNA was harvested and purified using Trizol® Reagent (Invitrogen, USA) and the quality was determined using an Agilent 2100 Bioanalyzer with RNA 6000 labchip kit (Agilent Technologies, USA). Experiments were performed and analyzed as a service at Welgene lnc. The statistical significance of the expression data was determined using fold change. Pathway analyses were performed using Ingenuity Pathway Analysis (IPA) software. Statistical significance of the expression data was determined using z-score and P-value.

### Analysis of proliferation and colony formation

The stable transfectant pEGFP-vector, pEGFP-GSKIP, vehicle (SH-SY5Y), GSKIP-KO#1, GSKIP-KO#7 and GSKIP-KO#9 cells were plated on 96-well plates in DMEM/F12 medium and incubated at 37 °C with 5% CO_2_. Then, 10 μl of the cell counting Kit-8 (CCK-8, Targetmol, Shanghai, China) solution was added to each well at four or five-time points and incubated for 1 h. The change in absorbance at 450 nm was measured photometrically and the experiment was repeated three times.

Cell survival was measured by a colony formation assay. Cells were seeded in 6-well plates at a density of 1000 cells per well for pEGFP-vector, pEGFP-GSKIP, vehicle (SH-SY5Y), GSKIP-KO#1, GSKIP-KO#7, and GSKIP-KO#9 cells, and allowed to grow for 10–14 days until visible colonies formed. Colonies were fixed with methyl alcohol and stained with crystal violet before the absorbance was measured at 570 nm to calculate the colony formation rate.

### Cell motility assays

Migration assays were conducted in 24-well hanging cell culture inserts (PIEP 12R 48; Millipore, St. Louis, MO, USA). Cells resuspended in 300 μl serum-free medium were added to the top chamber (1 × 10^5^ cells/well) and medium supplemented with Dmem/F12 medium supplemented with 10% FBS was added to the bottom chamber as a chemoattractant. Following 16–18 h incubation at 37 °C, cells that migrated through the membrane (migration) were fixed and stained with 0.1% crystal violet (Sigma-Aldrich). The number of cells was counted in three random fields under 100 × objective lens.

Cell migration was determined by the wound healing assay using IBIDI Culture Inserts (IBIDI GmbH, Martinsried, Germany). Cells (3 × 10^5^ cells/70μL/well) were seeded into each well of a 24 well with a culture insert (Ibidi GmbH, Martinsried, Germany). Cell debris was removed by washing with PBS, and the cells were cultured in media. Images were captured after wounding and the distance migrated by the cell monolayer to close the wounded area at 0, 24, and 36 h was measured. The wound closure rate was equal to the recovered distance divided by the original width of the scratch.

### Statistical analysis

Data are presented as mean ± standard deviation. Statistical analyses were performed using one-way analysis of variance. Data were compared using the student’s t-test, with a significant difference set at *p < 0.05, **p < 0.01, ***p < 0.001.

## Results

### GSKIP promotes the proliferation of SH-SY5Y cell clones, and the inhibition of GSK3β activity prevents SH-SY5Y cell clone differentiation in response to RA

**Fig. 1:**
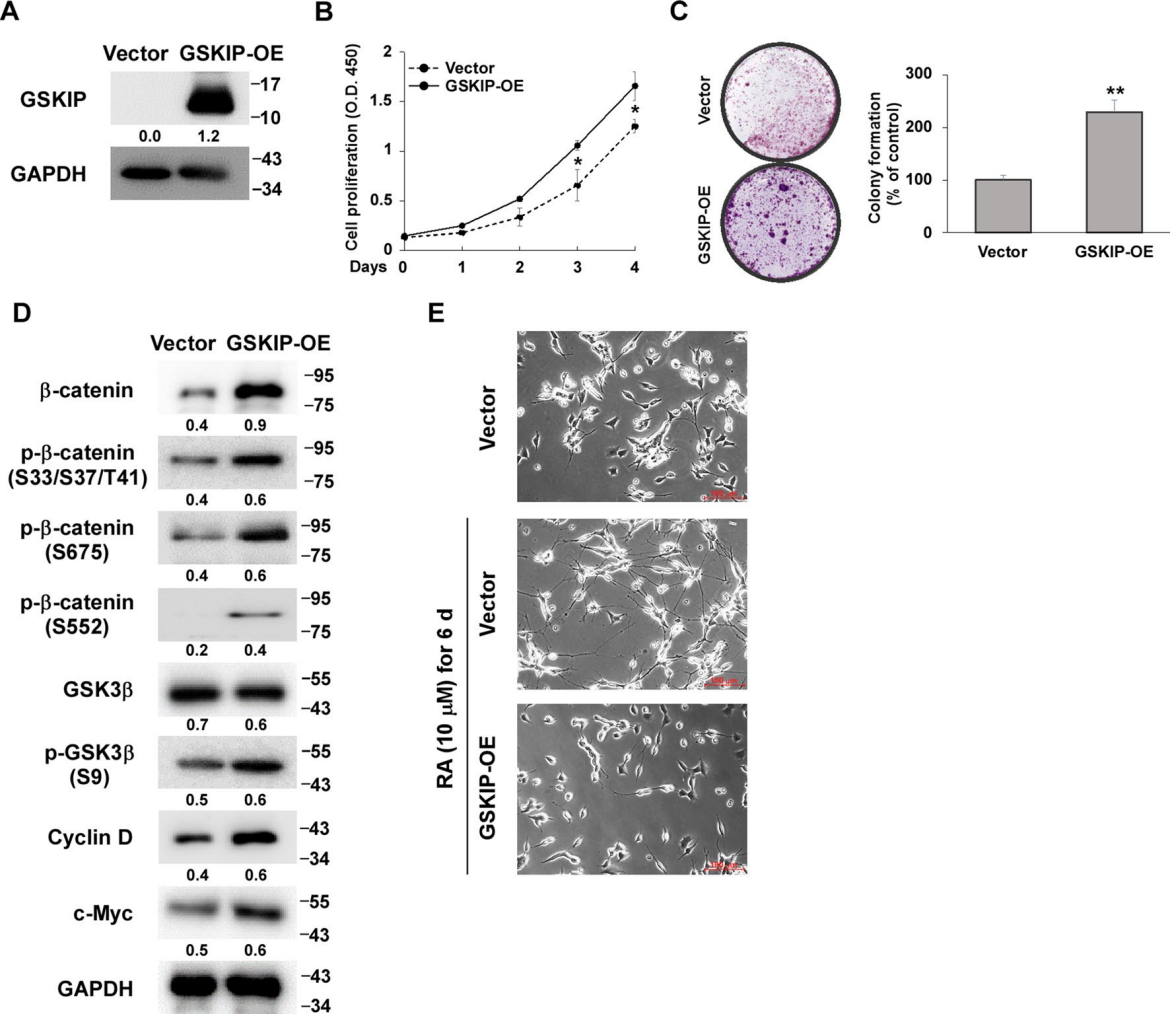
GSKIP promotes SH-SY5Y cell proliferation and inhibition of GSK3β activity prevents SH-SY5Y neuroblastoma cell differentiation in response to retinoic acid (RA) treatment.  **A** Western blot verification of GSKIP overexpression in SH-SY5Y cells. **B** Cell proliferation and **C** colony formation of SH-SY5Y cells upon GSKIP overexpression. **D** GSKIP overexpression promoted cell cycle progression via the GSK3β/β-catenin pathway. **E** Overexpression of GSKIP inhibited differentiation of SH-SY5Y cells in response to RA treatment. The bar graph represents the mean of triplicates ± SD. *p < 0.05, **p < 0.01, ***p < 0.001 compared to the Vehicle group

Our previous study revealed that GSKIP overexpression (GSKIP-OE) could induce cell cycle progression by increasing the accumulation of β-catenin in the nucleus and downregulating the association of β-catenin and N-cadherin in the membrane. GSKIP affects the transcriptional state of the cell in neuron development and neuron outgrowth by regulating the functional interplay of the GSK3β/β-catenin, β-catenin/Cyclin D1, and β-catenin/N-cadherin pools during RA treatment in SH-SY5Y cells. Therefore, we revisited how GSKIP-OE regulated β-catenin and N-cadherin during RA treatment, reintroducing GSKIP into SH-SY5Y cells and verifying the OE status of GSKIP by western blotting (Fig. 1A). Subsequently, we demonstrated that GSKIP-OE could promote cell proliferation and cell growth (Fig. 1B, C). To further determine which phosphorylation status of β-catenin is involved in translocation to the nucleus, phosphor-β-catenin (S33/S37/T41) as a β-catenin active form and both phosphor-β-catenin (S675) and β-catenin (S552) as β-catenin stable forms were examined. GSK3β (S9) was used for β-catenin phosphorylation for subsequent β-catenin degradation. GSKIP-OE increased β-catenin (S33/S37/T41) with increased p-GSK3β (S9) for β-catenin degradation, implying an unstable status and no role in translocation into the nucleus. However, the levels of phosphor-β-catenin (S675) and β-catenin (S552) also increased, and both were considered stable forms of β-catenin via the PKA site for translocation into the nucleus. Moreover, Cycle D and c-Myc also increased under GSKIP-OE (Fig. 1D) and GSKIP-OE inhibited neuron differentiation of SH-SY5Y cells in response to RA treatment (Fig. 1E). Therefore, GSKIP-OE could induce cell proliferation rather than differentiation through the regulation of GSK3β/β-catenin pathways under RA treatment. Notably, phosphor-β-catenin (S675) and β-catenin (S552) are involved in the translocation into the nucleus in the context of GSKIP-OE in SH-SY5Y cells.

### Generation and validation of SH-SY5Y cell clones with targeted deletions in GSKIP using CRISPR/Cas9 in SH-SY5Y cell clones

**Fig. 2:**
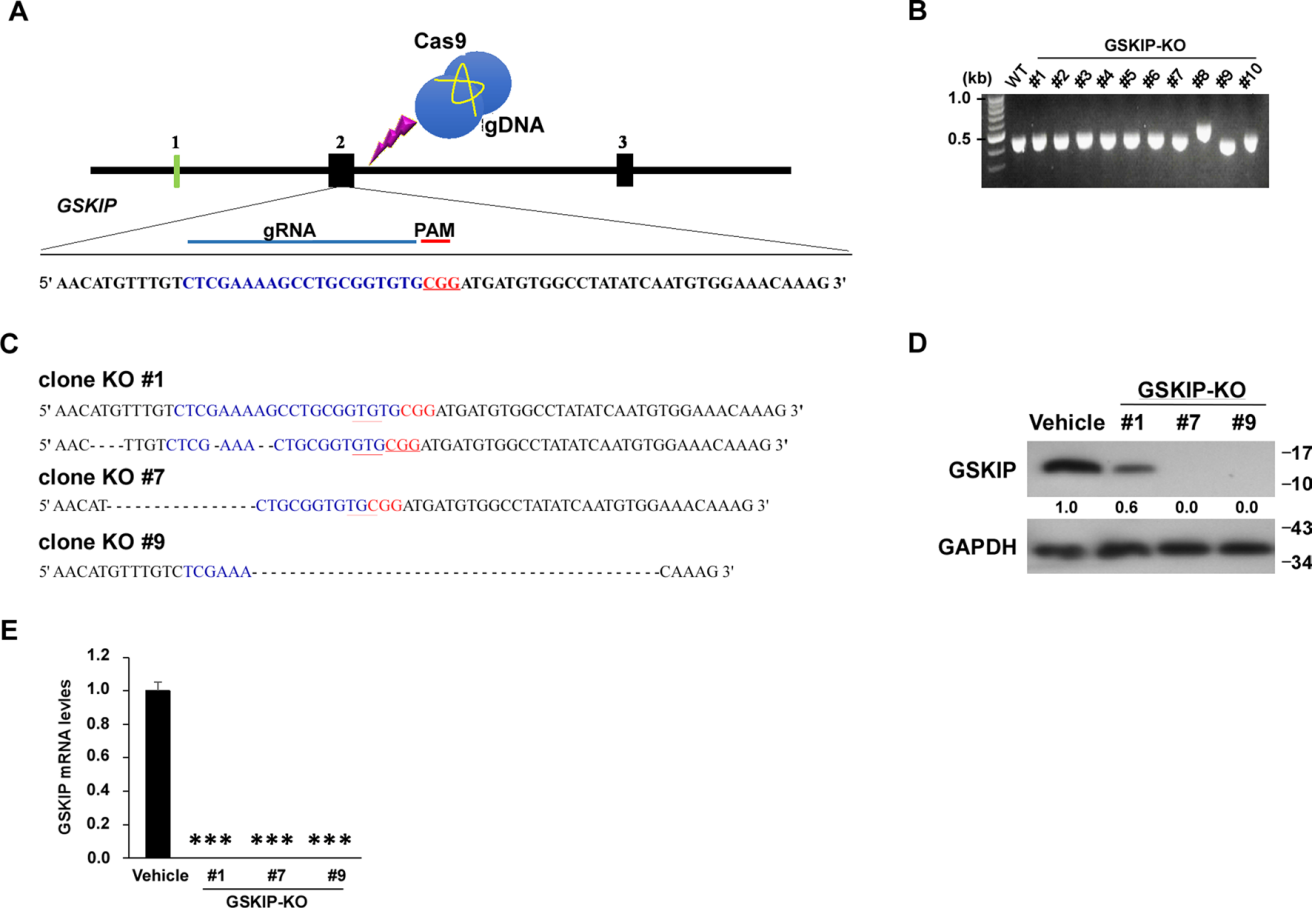
Generation and validation of SHSY-5Y cell clones with targeted deletions in GSKIP using CRISPR/Cas9. **A** Schematic representation of the genomic DNA structure of GSKIP with exons numbered. The sequence including CGG PAM targeted by gRNA in the CRISPR-Cas9 system is shown in red. **B** Relative telomere lengths were detected by RT-PCR in the GSKIP knockout cell line and the control. **C** Clones #1, 7, and 9 had the highest degree of GSKIP reduction and were sequenced and aligned with the wildtype sequence (− : deleted bases). **D** Western blot analysis of knockout cell clones to evaluate the expression of GSKIP in SHSY-5Y cells. GAPDH was used as a loading control. **E** qPCR analysis of knockout cell clones to evaluate the GSKIP expression in SHSY-5Y cells. 18S was used as a loading control. The bar graph represents the mean of triplicates ± SD. *p < 0.05, **p < 0.01, ***p < 0.001 compared to the vehicle group

To further investigate how GSKIP functions in and modulates neuron outgrowth, a CRISPR/Cas9 system was used to knockout the GSKIP gene and determine the function. The GSKIP knockout (GSKIP-KO) sequence included CGG PAM targeted by gRNA in a CRISPR Cas9 system, as shown in Fig. 2A. RT-PCR was performed to detect relative telomere lengths of the GSKIP-KO cell lines and control (Fig. 2B). Table 1 lists the number of clones loss base at the right locus and the number of clones carrying mutations leading to the loss of a functional allele (KO). Since #6 is an in-frame deletion (#1, #7, and #9 are out-frame deletion), #1, #7, and #9 were selected for further experiments. These cell clones (#1, #7, and #9) presented with the highest degree of GSKIP reduction for sequencing and aligning with the WT sequence (− : deleted bases, Fig. 2C). Western blotting indicated that GSKIP was not expressed in #7 and #9 and only slightly expressed in #1 (Fig. 2D and Figure S1), also #7 and #9 cell clones are homozygous deletions and #1 is a heterozygous deletion (Figure S2). qPCR analysis revealed that GSKIP was not expressed in these three cell clones (Fig. 2E), therefore, three GSKIP-KO cell clones with targeted deletions of GSKIP were generated using CRISPR/Cas9.

**Table 1:** SHSY-5Y cell clones with targeted deletions in GSKIP using CRISPR/Cas9. The number of clones loss base at the right locus, and the number of clones carrying mutations leading to the loss of a functional allele (knockout)

SH-SY5Y	PCR Product (bp)	Loss base	Stop codon	Protein length (AA)	kDa (× 110)
Vehicle	440	–	–	139	15.3
clone KO#1	430	WT/7 bp 51^th^ AA	Out of frame	77	8.5
clone KO#7	424	16 bp 51th AA	Out of frame 73^th^	72	7.9
clone KO#9	397	43 bp 56th AA	Out of frame 64th	63	6.9

### Knockout of GSKIP expression enhances cell aggregation in SH-SY5Y cell clones

**Fig. 3:**
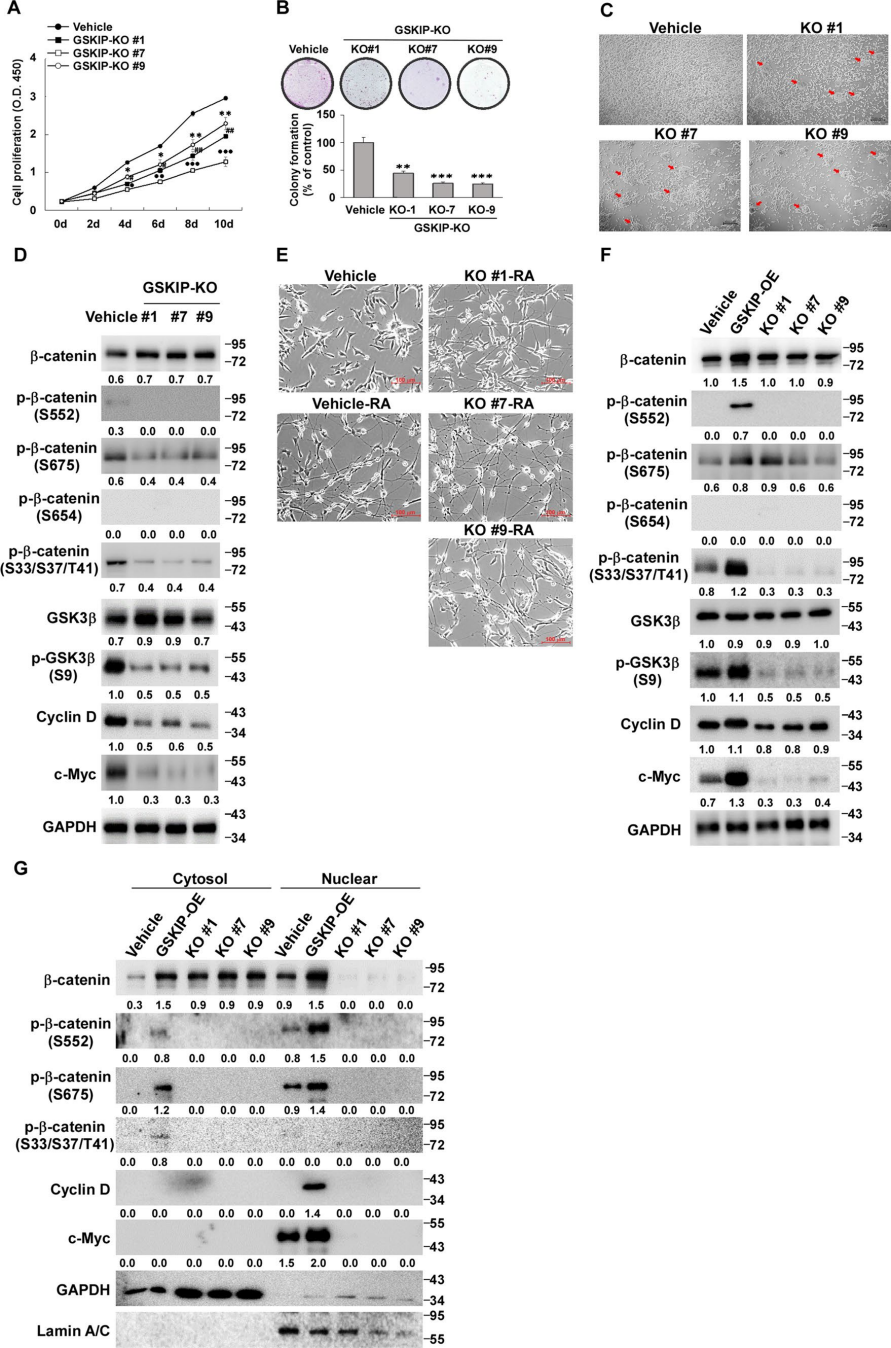
Knockout of GSKIP expression enhances cell–cell aggregation. **A** Cell proliferation was assessed by the MTT assay and measuring the absorbance at 570 nm. **B** Colony formation of SH-SY5Y cells upon GSKIP knockout. **C** Cellular morphology of SH-SY5Y, red arrows indicates cell aggregates formed over time. Scale bar = 200 μM. **D** GSKIP knockout inhibited cell cycle progression via the GSK-3/β-catenin pathway. **E** Knockout of GSKIP promoted differentiation of SH-SY5Y cells in response to RA treatment. **F** GSKIP overexpression and knockdown suppression and promoting cell cycle progression via the GSK-3/β-catenin pathway. **G** Western blot analysis of cytoplasmic and nuclear GSK-3/β-catenin pathway upon GSKIP knockout or overexpression. GAPDH was used as the internal control for the cytoplasmic proteins, whereas LaminA/C was used as the internal control for the nuclear proteins. The bar graph represents the mean of triplicates ± SD. *p < 0.05, **p < 0.01, ***p < 0.001 versus control (GSKIP-KO #1); #*p* < 0.05, ##*p* < 0.01, ###*p* < 0.001 versus control (GSKIP-KO #7); ⁕*p* < 0.05, ⁕⁕*p* < 0.01, ⁕⁕⁕*p* < 0.001 versus control (GSKIP-KO #9); B group *p < 0.05, **p < 0.01, ***p < 0.001 compared to the vehicle group

The three GSKIP-KO cell clones (#1, #7, and #9) were used to further investigate the function of GSKIP in neuron outgrowth, demonstrating that cell proliferation was suppressed (Fig. 3A) and cell growth decreased in these three clones (Fig. 3B). Interestingly, the GSKIP-KO cell clones exhibited aggregation behavior (Fig. 3C, red arrows indicate examples), phosphor-β catenin (S33/37/41), β-catenin (S675), and β-catenin (S552) were all attenuated and the levels of GSK3β (S9), Cyclin D, and c-Myc also decreased (Fig. 3D). The GSKIP-KO cell clones still promoted differentiation (neuron outgrowth) under RA treatment (Fig. 3E). Furthermore, GSKIP could upregulate phosphor-β catenin (S33/37/41), β-catenin (S675), and β-catenin (S552) and also positively modulate Cyclin D and c-Myc (Fig. 3F). It is interesting that β-catenin (S33/S37/T41) was not accumulated in nucleus sites, whereas β-catenin (S675) and β-catenin (S552) were partially accumulated in nuclear sites, indicating that only phosphor-β-catenin (S675) and β-catenin (S552) could stabilize β-catenin and translocate into the nucleus for further downstream activation (Fig. 3G). Taken together, these results demonstrate that GSKIP-KO cell clones enhance cell aggregation behavior, rather than cell differentiation, by suppressing GSK3β/β-catenin pathways and cell cycle progression in SH-SY5Y cells. In particular, phosphor-β-catenin (S675) and β-catenin (S552) were downregulated in the suppression of GSK3β/β-catenin pathways under GSKIP conditions in SH-SY5Y cells.

### Wnt signaling pathway and EMT/MET phenotype are associated with GSKIP-KO in SH-SY5Y cell clones

**Fig. 4:**
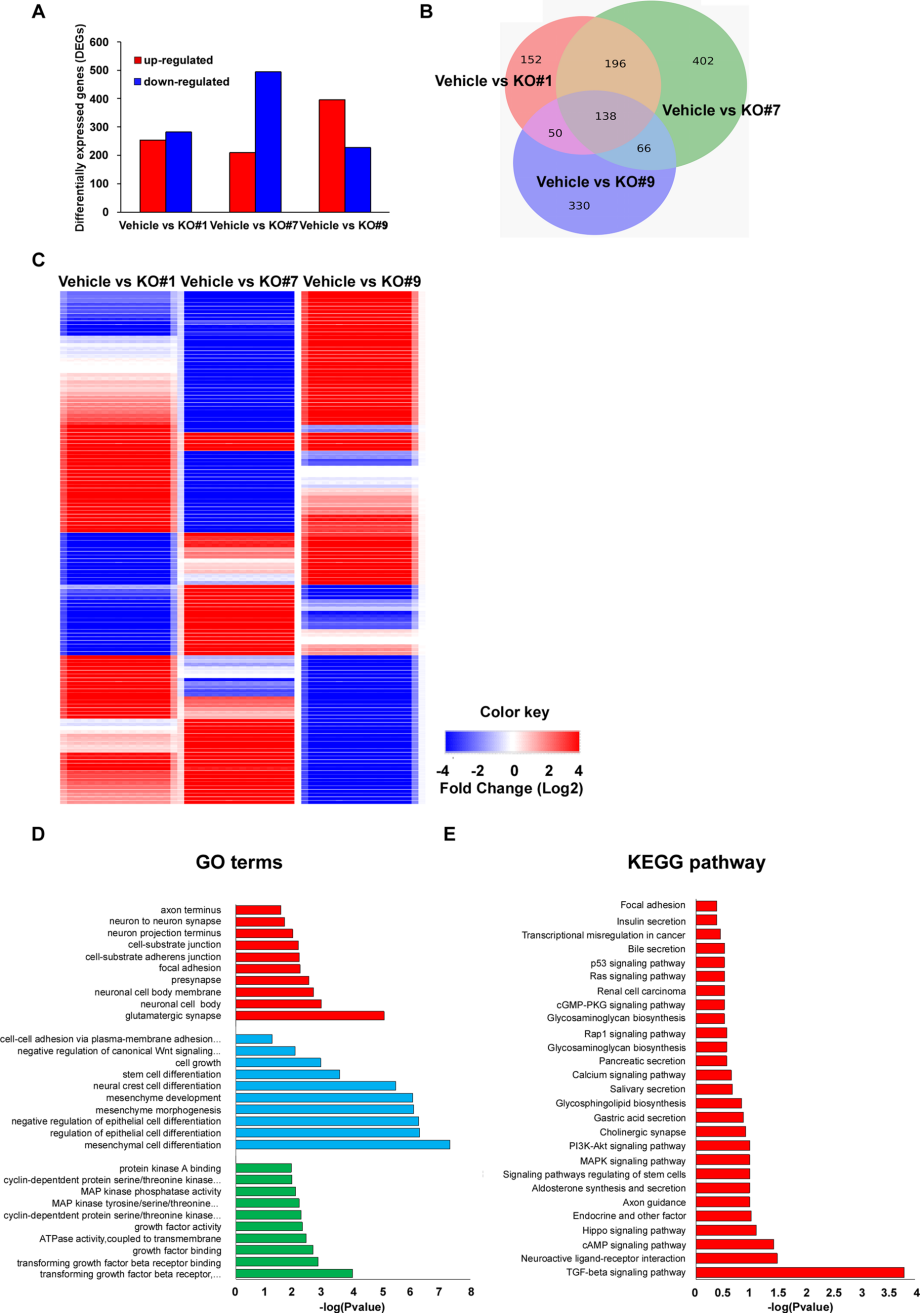
Venn diagram showing GO and KEGG pathway enrichment analysis of DEGs in GSKIP-KO cells. **A** Numbers of DEGs (≥ twofold difference; *p* < 0.05) in the Vehicle versus KO#1, Vehicle vs KO#7, and Vehicle versus KO#9. **B** Overlapping areas show the common DEGs between different sources of GSKIP-KO cells mRNA interactions. **C** Heatmap of log CPM values for the top 138 genes DE in WT versus GSKIP-KO cells. Samples with relatively high expression of a given gene are marked in red and samples with relatively low expression are marked in blue. Lighter shades and white represent genes with intermediate expression levels. **D** Top significantly enriched GO terms of down-and upregulated DEGs, including MF (Red), BP (Blue), and CC (Green). The x-axis represents the number of DEGs involved in GO terms, and the y-axis the significantly enriched GO terms. (E) KEGG pathway enrichment analysis of overlapping DEGs. The x-axis in terms of the significant KEGG pathway

Bioinformative tools were used to analyze the possible molecular mechanisms and potential genes involved in the cell aggregation phenotype. GSKIP-mRNA interactions were used to identify the common up- and downregulated differentially expressed genes (DEGs) in the vehicle with three different GSKIP-KO SH-SY5Y cell clones (#1, #7, and #9) (Fig. 4A). After data processing, 138 overlapping DEGs were identified within the three GSKIP-KO cell clones (Fig. 4B). Subsequently, the 138 genes in wildtype versus GSKIP-KO cells were displayed in a heatmap of log CPM values (Fig. 4C and Figure S2). GO and KEGG enrichment analysis revealed that GSKIP-KO is highly related to mesenchyme development and cell-substrate adherent junction functions, which are involved in the EMT/MET conversion phenotype (Fig. 4D), and cell adhesion pathways and the EMT/MET pathway are highly likely to play a role in the underlying mechanisms (Fig. 4E). In summary, cell adhesion signaling pathways and EMT/MET phenotypes are highly related to GSKIP-KO cell clones, and the Wnt/β-catenin signaling pathway and EMT/MET phenotype are associated with GSKIP-KO in SH-SY5Y cell clones.

### GSKIP modulates the interplay between EMT and MET in SH-SY5Y cells

**Fig. 5:**
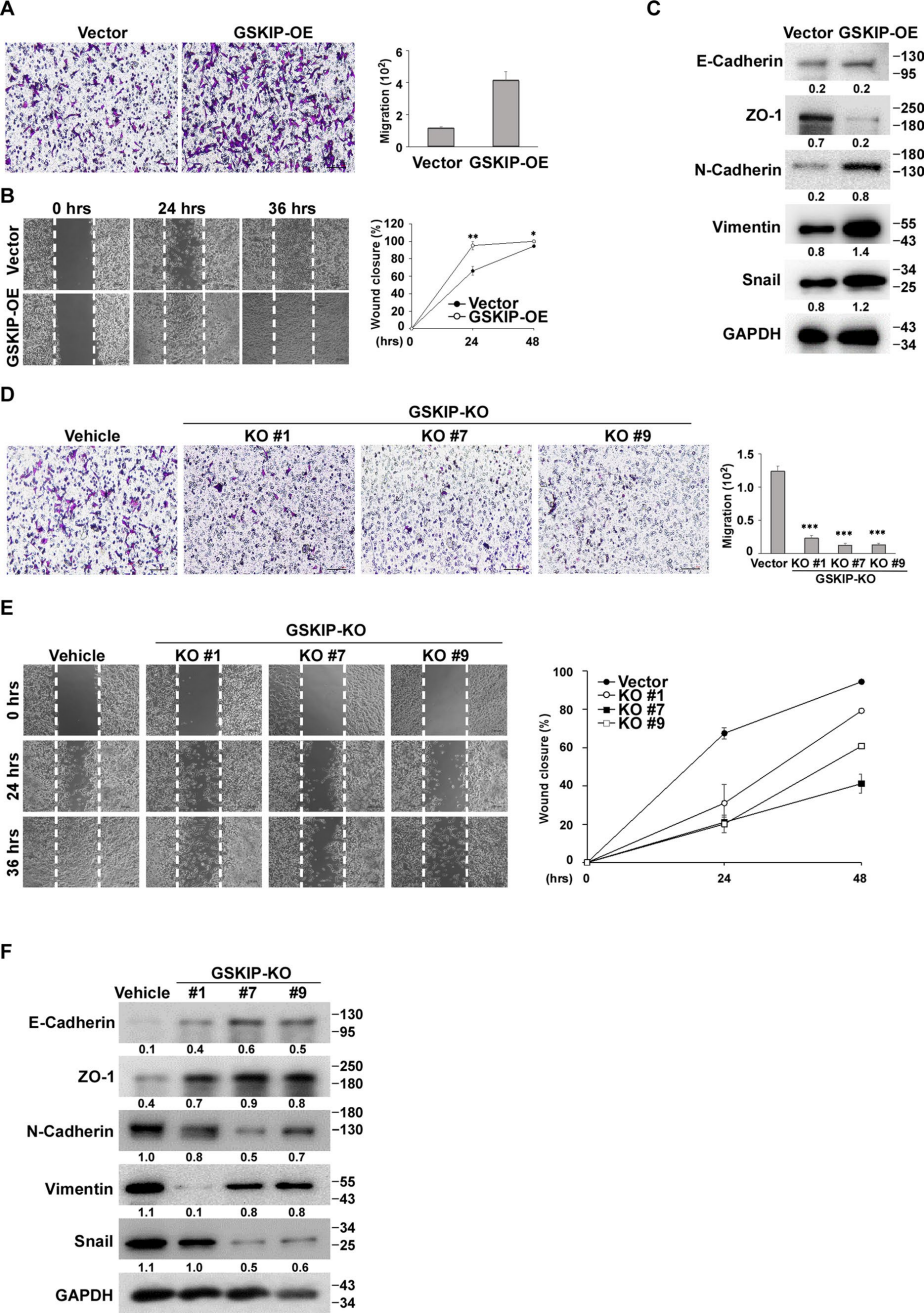
GSKIP modulates epithelial-to-mesenchymal transition (EMT) in SHSY-5Y cells. **A**, **D** Transwell cell migration assays (left panel, representative pictures of migration chambers; right panel, average counts from four random microscopic fields). **B**, **E** Cell motility was evaluated by the wound healing assay. Cells were plated in 2-well IBIDI chambers. After removing the insert, images were taken 0, 24, and 36 h after wound formation. Graphs show the closure rate. **C**, **F** The EMT marker expression regulation in modulated GSKIP gene expression SHSY-5Y cells. GAPDH was used as the internal control for the proteins. The bar graph represents the mean of triplicates ± SD. *p < 0.05, **p < 0.01, ***p < 0.001 compared to the vehicle group

The status of EMT and MET are involved in the initiation of metastasis, pluripotent stem cell reprogramming, and wound healing (Kalluri 2009; Pastushenko et al. 2018). Conversely, direct cell–cell contacts (cell aggregation) initiated and mediated by cell surface molecules or cell adhesion molecules, such as E-cadherin, N-cadherin, Snail, and vimentin (Chooi and Chew 2019), are considered MET processes. Traditionally, the loss of E-cadherin is considered to be a fundamental event in EMT in epithelial cells. However, N-cadherin replaces E-cadherin during neurulation and forms strong adherent junctions to maintain the tissue architecture of neural tissues as well as regulate the proliferation and differentiation of neural progenitor cells. Therefore, we examined the invasion and migration abilities of GSKIP-OE and the vehicle, revealing that GSKIP-OE cells exhibited a strong ability for cell migration and invasion (Fig. 5A, B). Western blotting indicated that the expression of N-cadherin, vimentin, and Snail were upregulated but not related to E-cadherin (Fig. 5C). Subsequently, the ability for invasion and migration of GSKIP-KO cell clones and the vehicle was examined, revealing that GSKIP-KO cells presented a low ability for invasion and migration compared to the vehicle and GSKIP-OE cells (Fig. 5D, E). Moreover, cell aggregation behavior was also detected in the three GSKIP-KO cell clones. The levels of N-cadherin, Snail, and vimentin decreased, whereas ZO-1 increased (Fig. 5F). Taken together, these results indicate that GSKIP could modulate the interplay with EMT and MET in SH-SY5Y cells. In particular, GSKIP-KO could promote MET which is involved in the cell aggregation phenotype through the regulation of N-cadherin in SH-SY5Y cells.

### Restored GSKIP expression in GSKIP-KO cell lines compensates for the loss of function in cell behavior and reverses MET in SH-SY5Y cell clones

**Fig. 6:**
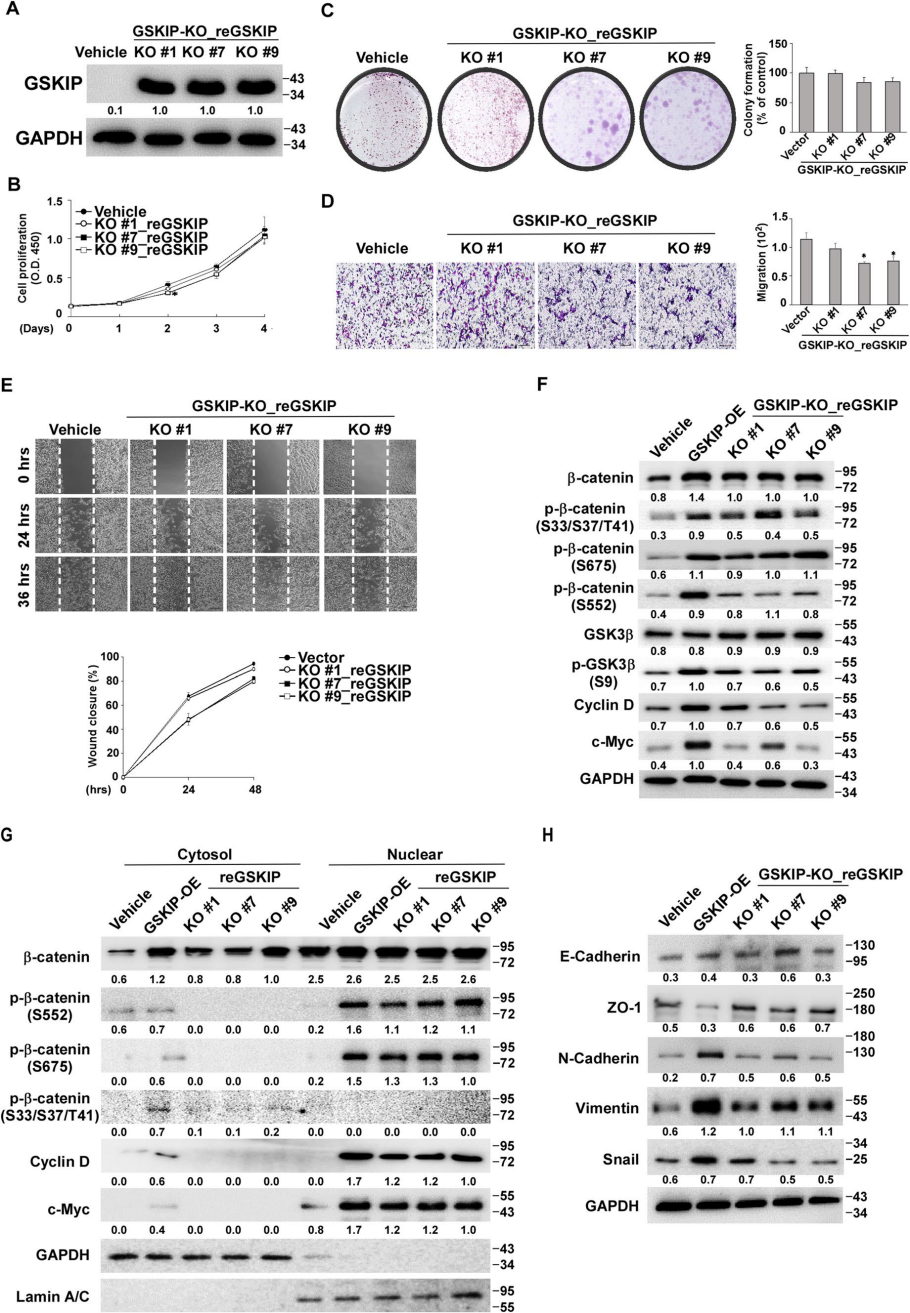
GSKIP modulates SH-SY5Y cell proliferation and migration via the GSK-3/β-catenin pathway. **A** Western blot analysis of knockout cell clones to evaluate the expression of GSKIP. **B** Cell proliferation, **C** colony formation, **D** cell migration, and **E** cell motility of knockout cell clones overexpressing GSKIP. **F** GSKIP knockout SH-SY5Y cells displayed increased protein expression of the GSK-3/β-catenin pathway when treated with overexpressed GSKIP. **G** GSKIP knockout SH-SY5Y cells displayed increased nuclear protein expression of the GSK-3/β-catenin pathway when treated with overexpressed GSKIP. **H** GSKIP knockout SH-SY5Y cells displayed increased protein expression for EMT marker expression regulation when treated with overexpressed GSKIP. GAPDH was used as the internal control for the proteins. The bar graph represents the mean of triplicates ± SD. *p < 0.05, **p < 0.01, ***p < 0.001 compared to the vehicle group

GSKIP was reintroduced into the three GSKIP-KO cell clones to determine whether GSKIP modulates the interplay with EMT and MET (GSKIP-KO_reGSKIP, Fig. 6A). Cell proliferation and cell growth increased in all three GSKIP-KO_reGSKIP cell clones (Fig. 6B, C) and these clones also regained their invasive and migratory abilities but no cell aggregation was observed (Fig. 6D, E). Additionally, the reintroduction of the GSKIP 130P mutant into GSKIP-KO cell clones could not restore the ability to promote cell proliferation (Figure S4). Phosphor-β-catenin (S33/S37/T41), GSK3β(S9), Cyclin D, and c-Myc were restored, as well as β-catenin (S675), and β-catenin (S552). However, only β-catenin (S675), and β-catenin (S552) translocated into the nucleus, not β-catenin (S33/S37/T41) (Fig. 6F, G). The expression of N-cadherin, Snail, and vimentin was increased in the three GSKIP-OE_reGSKIP cell clones but no E-cadherin was detected (Fig. 6H). Therefore, restoring GSKIP in GSKIP-KO cell clones could restore cell proliferation and growth and reverse MET in SH-SY5Y cells.

## Discussion

This study is the first to reveal the remarkable neuronal functions of GSKIP that modulate the subsequent status of EMT/MET. GSKIP plays a critical role in the phosphorylation of β-catenin for downstream activation. The overexpression of GSKIP upregulated not only phosphor-β-catenin (S33/S37/T41) but also β-catenin (S675) and β-catenin (S552), which translocate into the nucleus to promote cell proliferation and stabilize the N-cadherin/β-catenin complex (pool) signaling pathways for the EMT phenotype. Conversely, the knockout of GSKIP downregulated phosphor-β-catenin (S675) and β-catenin (S552) and attenuated N-cadherin/β-catenin complex (pool) signaling pathways to present the MET phenotype (cell aggregation). It was also demonstrated that the N-cadherin/β-catenin complex has a major role in the EMT/MET phenotype in tumorigenesis in neuron SH-SY5Y cells. Our study revealed new mechanisms through which GSKIP regulates the EMT/MET phenotype through the GSKIP/GSK3β/β-catenin/N-cadherin axis for new insights into the neuronal function and behavior of SH-SY5Y human neuroblastoma cells.

According to our previous study, the overexpression of GSKIP upregulates p-GSK3β (S9) in RA-mediated neuron outgrowth (Lin et al. 2009). Surprisingly, the knockout of GSKIP contributes to cell aggregation via the downregulation of p-GSK3β (S9), rather than the suppression of RA-mediated neuron outgrowth. GSKIP-KO modulates the interplay of EMT/MET. In general, EMT and its reverse process, MET, instantiate fundamental principles in a diverse range of physiological and pathological processes (Lai et al. 2020). The process is regulated or promoted at different levels by multiple factors, including cell signaling, transcriptional control epigenetic modification, and post-translational modifications, such as transforming growth factor-β (TGF-β), fibroblast growth factor family, and epidermal growth factor (EGF) (Williams et al. 2019). EMT is traditionally characterized by decreased E-cadherin expression (Sulaiman et al. 2018), and the loss of E-cadherin in cancer cells leads to the metastatic dissemination and activation of several EMT transcription factors. However, it has been argued that the loss of E-cadherin is neither causal nor necessary for EMT and that the restoration of E-cadherin expression in E-cadherin negative malignant cells did not reverse the EMT (Hollestelle et al. 2013). In contrast to E-cadherin, N-cadherin is prevalent in nonepithelial tissues and is expressed in various cells, such as neural cells, endothelial cells, stromal cells, and osteoblasts (Mrozik et al. 2018). In neuron tissue, N-cadherin serves as an indicator of ongoing EMT during neurulation and forms strong adherent junctions to maintain the tissue architecture of neural tissues, as well as regulates the proliferation and differentiation of neural progenitor cells (Miyamoto et al. 2015). These data demonstrated that GSKIP-KO downregulates N-cadherin to promote MET (cell aggregation) and reintroduction of GSKIP upregulates N-cadherin in SH-SY5Y cells. Other EMT/MET markers, such as Snail, vimentin, and ZO-1, exhibited the same pattern. Combined with our previous study of GSKIP-OE, we are the first to discover that GSKIP has dual functions for regulating neuron behaviors in context of GSKIP, the GSKIP-OE promotion of neuron proliferation and GSKIP-KO modulation of EMT/MET status.

E-cadherin or N-cadherin binds to neighboring cadherins through the extracellular domain, mediating cell–cell adhesion, preventing tumor cell migration, and preventing in vivo dissemination/invasiveness. The intracellular domain of E-cadherin or N-cadherin binds to β-catenin (an effector of Wnt signaling), thereby preventing the nuclear translocation of β-catenin and β-catenin/T-cell factor (TCF)-mediated transactivation and impedes Wnt signaling and the acquisition of mesenchymal traits (Li and Mattingly 2008). In general, cadherins are considered negative regulators of the Wnt pathway that sequesters β-catenin from TCF family transcription factors to the plasma membrane (Loh et al. 2019). Crosstalk between cadherins and the canonical Wnt/β-catenin pathway are linked in multiple ways and Wnt signaling has been shown to downregulate E-cadherin in embryonic mouse brains (Haÿ et al. 2012). Consistent with our previous study, GSKIP-OE increases the amount of β-catenin, resulting in the translocation of β-catenin to the nucleus under RA. Conversely, GSKIP-KO downregulates phosphor-β-catenin (S33/S37/T41), β-catenin (S675), and β-catenin (S552) through the reduction of p-GSK3β (S9) before reducing the level of Cyclin D and c-Myc in the nucleus. Collectively, GSKIP-KO reduced N-cadherin/β-catenin pools in cytoplasmic or nuclear sites through the degradation of β-catenin to present the MET (cell aggregation) phenotype.

The most well-known modifications of β-catenin are a series of phosphorylations that continually promote degradation of the cadherin-free pool or β-catenin pool. However, cadherin-mediated β-catenin and the phosphorylation status of β-catenin which modulates the process is still unclear. In general, β-catenin (S33/37/41) is viewed as a phosphorylated form of β-catenin through the suppression of GSK3β activity by GSK3β (S9), followed by ubiquitination and degradation. Unphosphorylated β-catenin in the cytosol migrates to and accumulates in the nucleus to bind TCF to activate downstream factors (Zhang and Wang 2020). β-catenin can exist in three distinct pools inside the cell, membranous, cytoplasmic, and nuclear (Kumar and Bashyam 2017), and interacts with E-cadherin at the cell membrane, playing a key structural role in the adherent junctions. β-catenin free in the cytoplasm is phosphorylated by the destruction complex for degradation and is then translocated to the nucleus where it contributes to the transcriptional regulation of genes. In this study, we examined several forms of phosphor-β-catenin, such as β-catenin (S33/S37/T41, GSK3β site), β-catenin (S675, PKA site), and β-catenin (S552, Akt site) (Brudvik et al. 2011; Fang et al. 2007; Liu et al. 2002; Sorrenson et al. 2021; Taurin et al. 2006). Surprisingly, our results revealed that phosphor-β-catenin (S33/S37/T41), β-catenin (S675), and β-catenin (S552) were all increased, indicating that phosphor-β-catenin (S675) and β-catenin (S552) were stable forms and could be involved in the translocation into the nucleus under GSKIO-OE. By contrast, under GSKIP-KO conditions, only phosphor-β-catenin (S675) and β-catenin (S552) were involved in the translocation to the nucleus to activate TCF activity in the context of GSKIP (Figs. 3G and 6G, lanes 2 and 3). Furthermore, high β-catenin/TCF activity can drive cell proliferation during tumor formation by activating the cell cycle regulators Cyclin D and c-Myc. Our results also demonstrated that Cyclin D and c-Myc decreased under GSKIP-KO and were restored when GSKIP was reintroduced. This is consistent with the general principle that phosphor-β-catenin (S675) and β-catenin (S552) are translocated into the nucleus for further cell cycle processes (Fig. 6G). It is not clear how GSKIP-OE regulates N-cadherin expression in SH-SY5Y cells, but our data suggest that GSKIP increases the amount of phosphor-b-catenin and N-cadherin expression resulting in the translocation of β-catenin (S673 and S552, but not S33/S37/T41) to the nucleus (Fig. 6F–H). The limitation of present study is that the similar experimental design should be confirmed under animal model for potential new drug in neuroblastoma treatment.

## Conclusions

**Fig. 7:**
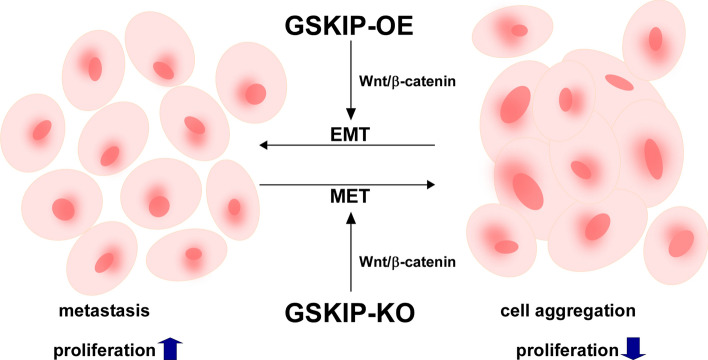
Involvement of GSKIP in potential signaling pathways affecting SHSY-5Y cell aggregation. Under the GSKIP-KO condition, SH-SY5Y cells appear to promote an aggregation phenotype. Under GSKIP-OE, SH-SY5Y could promote proliferation via the EMT/MET pathway to enable metastasis. *OF* overexpression; *KO* knockout; *EMT/MET* epithelial mesenchymal transition/mesenchymal epithelial transition

Regardless of GSKIP involved in differentiation under RA treatment (Figure S4), our data suggest the working model depicted in Fig. 7. Under the GSKIP-KO condition, SH-SY5Y human neuroblastoma cells have an aggregation phenotype as MET via the Wnt/β-catenin/GSK3β/Cyclin D/c-Myc pathways promotes cell survival in harsh environments. Under GSKIP-OE, SH-SY5Y promotes cell proliferation as EMT via the Wnt/β-catenin/GSK3β/Cyclin D/c-Myc pathway to promote metastasis. In combination with our previous experiments, GSKIP-OE and under RA treatment could induce neuron outgrowth for a differentiation phenotype, with phosphor-β-catenin (S33/37/41) having a major role in this pathway. However, GSKIP-KO is involved in EMT/MET signaling for an aggregation phenotype. Phosphor-β-catenin (S675) and β-catenin (S552) interplay and translocate into the nucleus for further EMT/MET processes. In conclusion, GSKIP plays a critical role in the interplay with EMT/MET, and thus may be a potential new target for neuroblastoma treatment.

## Data Availability

All data generated or analysed during this study are included in this published article.

## Abbreviations

GSK3β: Glycogen synthase kinase-3β
GSKIP: GSK3β interacting protein
AKAP: A-kinase anchor protein
GSKIP-KO: Knock out GSKIP
RA: Retinoic acid
EMT/MET: Epithelial mesenchymal transition/mesenchymal epithelial transition
PKA: Protein kinase A
Drp1: Dynamin-related protein 1
WT: With wildtype
OE: Overexpression
DEGs: Differentially expressed genes
TGF-β: Transforming growth factor-β
TCF: T-cell factor
NGS: Next-generation sequencing
IPA: Ingenuity Pathway Analysis
